# A comparison of direct versus self-report measures for assessing physical activity in adults: a systematic review

**DOI:** 10.1186/1479-5868-5-56

**Published:** 2008-11-06

**Authors:** Stéphanie A Prince, Kristi B Adamo, Meghan E Hamel, Jill Hardt, Sarah Connor Gorber, Mark Tremblay

**Affiliations:** 1 Department of Population Health, University of Ottawa, Ottawa, Ontario, Canada; 2 Healthy Active Living and Obesity Research Group, Children's Hospital of Eastern Ontario Research Institute, Canada; 3 Faculty of Health Science, School of Human Kinetics, University of Ottawa, Canada; 4 Division of Cancer Care and Epidemiology, Cancer Research Institute, Department of Community Health and Epidemiology, Queen's University, Kingston, Ontario, Canada; 5 Health Information and Research Division, Statistics Canada, Ottawa, Ontario, Canada; 6 Physical Health Measures Division, Statistics Canada, Ottawa, Ontario, Canada

## Abstract

**Background:**

Accurate assessment is required to assess current and changing physical activity levels, and to evaluate the effectiveness of interventions designed to increase activity levels. This study systematically reviewed the literature to determine the extent of agreement between subjectively (self-report e.g. questionnaire, diary) and objectively (directly measured; e.g. accelerometry, doubly labeled water) assessed physical activity in adults.

**Methods:**

Eight electronic databases were searched to identify observational and experimental studies of adult populations. Searching identified 4,463 potential articles. Initial screening found that 293 examined the relationship between self-reported and directly measured physical activity and met the eligibility criteria. Data abstraction was completed for 187 articles, which described comparable data and/or comparisons, while 76 articles lacked comparable data or comparisons, and a further 30 did not meet the review's eligibility requirements. A risk of bias assessment was conducted for all articles from which data was abstracted.

**Results:**

Correlations between self-report and direct measures were generally low-to-moderate and ranged from -0.71 to 0.96. No clear pattern emerged for the mean differences between self-report and direct measures of physical activity. Trends differed by measure of physical activity employed, level of physical activity measured, and the gender of participants. Results of the risk of bias assessment indicated that 38% of the studies had lower quality scores.

**Conclusion:**

The findings suggest that the measurement method may have a significant impact on the observed levels of physical activity. Self-report measures of physical activity were both higher and lower than directly measured levels of physical activity, which poses a problem for both reliance on self-report measures and for attempts to correct for self-report – direct measure differences. This review reveals the need for valid, accurate and reliable measures of physical activity in evaluating current and changing physical activity levels, physical activity interventions, and the relationships between physical activity and health outcomes.

## Background

Physical activity is defined as "any bodily movement produced by the skeletal muscle that results in energy expenditure (EE)" [1]. Inactivity is known to be associated with an increased risk for many chronic diseases including: coronary artery disease, stroke, hypertension, colon cancer, breast cancer, Type 2 diabetes, and osteoporosis [2], as well as premature death. The economic burden of physical inactivity in Canada has been estimated to be $2.1 billion [2]. Physical activity levels are often monitored to assess the health behaviours of the population and their association with health status including mortality and morbidity rates. Accurate assessment of physical activity is required to identify current levels and changes within the population, and to assess the effectiveness of interventions designed to increase activity levels.

Data collection at the population level often involves self-report (subjective) measures of physical activity through the use of questionnaires, diaries/logs, surveys, and interviews. These measures are frequently used due to their practicality, low cost, low participant burden, and general acceptance [3]. Although self-reports are useful for gaining insight into the physical activity levels of populations, they have the capacity to over- or underestimate true physical activity energy expenditure and rates of inactivity. The self-report methods are often wrought with issues of recall and response bias (e.g. social desirability, inaccurate memory) and the inability to capture the absolute level of physical activity.

As self-report methods possess several limitations in terms of their reliability and validity [4], objective or direct measures of physical activity are commonly used to increase precision and accuracy and to validate the self-report measures. Direct measures are believed to offer more precise estimates of energy expenditure and remove many of the issues of recall and response bias. Direct measures consist of calorimetry (i.e., doubly labeled water, indirect, direct), physiologic markers (i.e., cardiorespiratory fitness, biomarkers), motion sensors and monitors (i.e., accelerometers, pedometers, heart rate monitors), and direct observation. Despite the advantages of using direct methods, these types of measures are often time and cost intensive and intrusive rendering them difficult to apply to large epidemiologic settings. These measures also require specialized training and the physical proximity of the participant for data collection. In addition, direct measures each possess their own limitations and no single "gold standard" exists for measuring physical activity or assessing validity [3].

The appropriate method for measuring physical activity at various levels depends on factors such as the number of individuals to be monitored, the time period of measurements and available finances [5]. Many previous studies have examined the reliability and validity of various self-report and direct methods for assessing physical activity. Results from these studies have been conflicting. To our knowledge no attempt has been made to synthesize the literature to determine the validity of physical activity measures in adult populations.

The primary objective of this study was to perform a systematic review to compare self-report versus direct measures for assessing physical activity in observational and experimental studies of adult populations. The results from this systematic review provide a comprehensive summary of past research and a comparison between physical activity levels based on direct versus self-report measures in adult populations.

## Methods

### Study criteria

The review sought to identify all studies (observational or experimental) that presented a comparison of self-report and direct measurement results to reveal differences in physical activity levels based on measurement in adult populations (18 years and over). Studies which examined only a self-report or direct measure, but not both were not included in the review. All study designs were eligible (e.g. retrospective, prospective, case control, randomized controlled trial, etc.) and both published (peer-reviewed) and unpublished literature were examined.

Only studies involving adult populations with a mean age of 18 years and older were considered. Abstracts and titles were examined for their mention of adult populations (using adult$.tw.), but the search relied mostly on the subject headings for adult age groups (exp adult/). This systematic review was conducted simultaneously with a systematic review of the same focus in child populations (mean age < 19 years). A separate pediatric review was carried out as a result of differences in measurement methodologies and hypothesized cognitive and recall abilities between adults and children [6].

The eligible self-report measures of physical activity included: diaries or logs; questionnaires; surveys; and recall interviews. Proxy-reports were excluded because they present issues of reliability due to the potential heterogeneity of reporters (e.g., spouse, trainer, coach, parent, caregiver). The eligible direct measures of physical activity included: doubly-labeled water (DLW), indirect or direct calorimetry, accelerometry, pedometry, heart rate monitoring (HRM), global positioning systems, and direct observation. Although no language restrictions were imposed in the search, only English language articles were included in the review. Abstracts were included if they provided sufficient details to meet inclusion criteria.

### Search strategy

The following electronic bibliographic databases were searched using a comprehensive search strategy to identify relevant studies reporting the use of both self-report and direct measures for assessing individual physical activity levels: Ovid MEDLINE(R) (1950 to April Week 4 2007); Ovid EMBASE (1980 to 2007 Week 18); Ovid CINAHL (1982 to April Week 4 2007); Ovid PsycINFO (1806 to April Week 1 2007); SPORTDiscus (1830 to April 2007); Physical Education Index (1970 to April 2007); Dissertations and Theses (1861 to April 2007); and Ovid MEDLINE (R) Daily Update (May 4, 2007). The search strategy is illustrated using the MEDLINE search as an example (Table 1) and was modified according to the indexing systems of the other databases. The OVID interface was used to search MEDLINE, EMBASE, CINAHL, and PsycINFO; Ebscohost was used to search SPORTDiscus; Scholar's Portal was used to search Physical Education Index; and ProQuest for Dissertations and Theses. Grey literature (non-peer reviewed works) included published abstracts and conference proceedings, published lists of theses and dissertations, and government reports. Knowledgeable researchers in the field were solicited for key studies of interest. The bibliographies of key studies selected for the review were examined to identify further studies.

**Table 1:** Medline search strategy

1	self report$.tw.
2	questionnaire$.tw.
3	diary.tw.
4	log$.tw.
5	survey.tw.
6	interview$.tw.
7	recall.tw.
8	physical activity assessment.tw.
9	Minnesota Leisure Time Physical Activity Questionnaire.tw.
10	Framingham Leisure Time Physical Activity Questionnaire$.tw.
11	Global Physical Activity Questionnaire$.tw.
12	(International Physical Activity Questionnaire or IPAQ$).tw.
13	(Godin Leisure Time Exercise Questionnaire or Godin-Shephard Leisure Time Exercise Questionnaire$).tw.
14	seven day physical activity recall$.tw.
15	(Bouchard three day physical activity record or bouchard 3 day physical activity record$).tw.
16	Baecke Questionnaire of Habitual Physical Activity.tw.
17	Modified Baecke Questionnaire for Older Adults$.tw.
18	Modifiable Activity Questionnaire$.tw.
19	Aerobics Center Longitudinal Study Physical Activity Questionnaire.tw.
20	CARDIA Physical Activity Questionnaire$.tw.
21	(Health Insurance Plan of New York Activity Questionnaire or HIP activity questionnaire).tw.
22	(physical activity questionnaire of the kuopio ischemic heart disease study or physical activity questionnaire of the KIHD study).tw.
23	Paffenbarger Physical Activity Questionnaire$.tw.
24	Lipid Research Clinics Questionnaire.tw.
25	Stanford Physical Activity Questionnaire$.tw.
26	Tecumseh Occupational Physical Activity Questionnaire$.tw.
27	Physical Activity Scale for the Elderly.tw.
28	Canada Fitness Survey$.tw.
29	(The MONICA Optional Study of Physical Activity or MOSPA$).tw.
30	Framingham Physical Activity Index.tw.
31	YALE Physical Activity Survey$.tw.
32	Zutphen Physical Activity Questionnaire$.tw.
33	or/1–32
34	(doubly labeled water or doubly labelled water).tw.
35	calorimet$.tw.
36	heart rate monitor$.tw.
37	acceleromet$.tw.
38	pedomet$.tw.
39	activity monitor$.tw.
40	CSA monitor.tw.
41	or/34–40
42	direct$ observ$.tw.
43	((direct$ or physical or objective) adj2 measur$).tw.
44	((reference or gold or criter$) adj standard$).tw.
45	(reliability or validity).tw.
46	or/42–45
47	physical activity.tw.
48	exercise.tw.
49	walk$.tw.
50	physical fitness.tw.
51	or/47–50
52	46 and 51
53	41 or 52
54	33 and 53
55	adult$.tw.
56	exp adult/
57	or/55–56
58	54 and 57

Two independent reviewers screened the titles and abstracts of all studies to identify potentially-relevant articles. Duplicates were manually removed. The full texts of all studies that met the inclusion criteria were then obtained and reviewed. When disagreements between reviewers occurred, consensus was achieved through discussion and/or with a third reviewer.

Standardized data abstraction forms were completed by one reviewer and verified by two others. Information was extracted on the type of study design, participant characteristics, sample size, and methods of physical activity measurement (self-report and direct measures employed, units of measurement, duration of direct measure, length of recall, and length of time between the self-report and directly measured estimates). Reviewers were not blinded to the authors or journals when extracting data.

### Risk of bias assessment

The Downs and Black [7] checklist was used to assess the risk of bias. The Downs and Black instrument was recommended for assessing risk of bias in observational studies in a recent systematic review [8] and other assessments [9] and was employed in this review to assess study quality including reporting, external validity, and internal validity (bias). The Downs and Black checklist consists of 27 items with a maximum count of 32 points. A modified version of the checklist was employed with items that were not relevant to the objectives of this review removed. The adapted checklist consisted of 15 items, including items 1–4, 6, 7, 9–13, 16–18, and 20 from the original list, with a maximum possible count of 15 points (higher scores indicate superior quality). The risk of bias assessment was carried out by two independent assessors and when disagreements between assessors occurred, consensus was achieved through discussion.

### Data synthesis

Percent mean difference was used as the main outcome of this analysis; it was calculated using the formula: [(self-report mean – direct mean)/direct mean]. Only studies with units of measurement that were the same for both the self-report and direct measures were used to calculate percent mean differences. Units were converted where possible. These studies were included in the direct comparison analyses. Forest plots (graphical displays of the percent mean differences across the individual studies) were constructed to present overall trends in agreement of physical activity by direct measure and gender. As most studies did not employ the same units of measurement (e.g. kcal/week, MET/day, MET-min/day) and did not report a measure of variance (e.g. standard deviations or standard errors), pooled estimates and confidence intervals were not calculated.

## Results

### Description of studies

**Figure 1:**
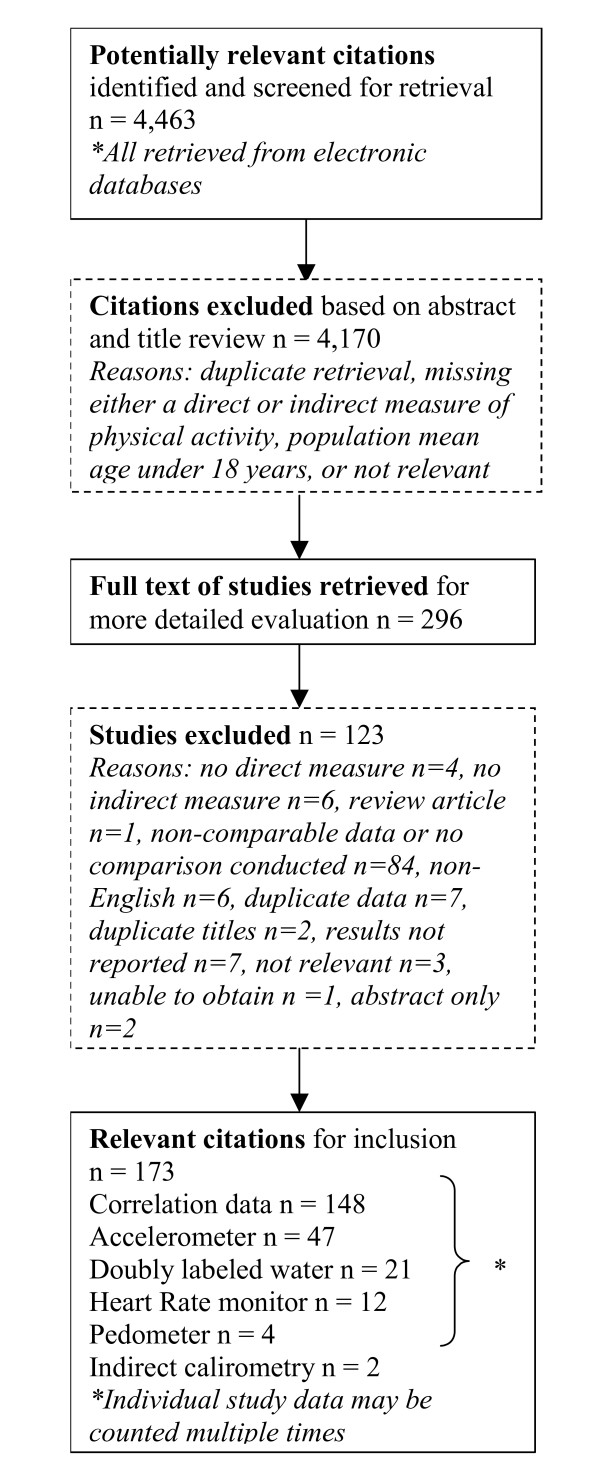
Results of the literature search.

The preliminary search of electronic bibliographic databases, reference lists and grey literature identified 4,463 citations (see Figure 1). Of these, 1,638 were identified in MEDLINE, 1,306 in EMBASE, 732 in CINAHL, 218 in PsycINFO, 133 in SportDISCUS, 34 in Physical Education Index, 3 in MEDLINE Daily Update, and 399 from Dissertations and Theses. After a preliminary title and abstract review, 296 full text articles were retrieved for a detailed assessment. Of these, 173 met the criteria for study inclusion. One hundred and forty-eight of these studies reported correlation statistics [10-157]. Seventy-four studies contained comparable data meaning the self-report and direct measurements were reported using the same units [11,15,17,19,20,23,32,33,44,48,53,56-59,65,73-77,80,88,90,92,94,100,102,105,111,114,116,119-121,128,131,134,135,138-140,143,148,151,153,154,158-183]. These studies were included in the direct comparison analyses and their characteristics are described in Table 2. Common reasons for excluding studies included: populations with mean ages less than 18 years, the absence of directly measured and self-report data on the same population, non-English language, duplicate reporting of data, and the absence of comparable units between measures or the absence of a direct comparison.

**Table 2:** Study and participant characteristics for studies with directly comparable data

**First Author – Year**	**Age Range or Mean (SD)**	**N_analyzed_**			**Population**	**Direct Measure**	**Indirect Measure**	**Units**	Testing Conditions*
		**Total**	**Men**	**Women**					
Adams 2003 A	40–65	80	0	80	Disease free	Accel	7-day PAR – Light	kcal/kg/day	A
Adams 2003 B						Accel	7-day PAR – MPA		
Adams 2003 C						Accel	7-day PAR – VPA		
Adams 2005 A	40–65	80	0	80	Disease free	DLW	7-day PAR 1	kcal/kg/day	C
Adams 2005 B						DLW	7-day PAR 2		
Adams 2005 C						DLW	24-hr PAR		
Ainsworth 1993 A	23–59	75	27	48	White collar workers	Accel	7-day PAR	average MET	NS
Ainsworth 1993 B						Accel	Tecumseh		
Ainsworth 1993 C						Accel	PAR		
Ainsworth 2000	20–60	50	0	50	Females	Accel	KPAS	MET-min/day	A
Atienza 2005	60.3 (9.1)	15	0	15	Filipino American	Accel	3-day PAD	min/3-day	A
Barnard 2002	22–59	15	8	7	Healthy volunteers	DLW	MAQ	MJ	NS
Bassett 2000	25–70	96	48	48	General population	Ped	CAQ	kcal/week	D
Bernstein 1998	35–69	41	18	23	General population	HRM	PAFQ	kcal/day	C
Bonnefoy 2001	66–82	19	19	0	Elderly	DLW	QAPSE	kcal/day	A
Boulay 1994 A	21 (5)	15	15	0	X-Country skiers	HRM	3-day PAD	MJ/day	C
Boulay 1994 B	22 (1)				Controls	HRM			
Buchowski 1999 A	20–50	115	45	70	Volunteers – Normal	Indirect cal.	PAD ≤ 2.4 METS	min/day	A
Buchowski 1999 B						Indirect cal.	PAD 2.5–4.4 METS		
Buchowski 1999 C						Indirect cal.	PAD ≥ 4.5 METS		
Buchowski 1999 D					Volunteers – Exercise	Indirect cal.	PAD ≤ 2.4 METS		
Buchowski 1999 E						Indirect cal.	PAD 2.5–4.4 METS		
Buchowski 1999 F						Indirect cal.	PAD ≥ 4.5 METS		
Clark 1994 A	37.3 (3.6)	14	0	14	Large eaters	DLW	PAD	MJ/day	C
Clark 1994 B	39.7 (2.0)				Small eaters	DLW			
Conway 2002 A	42 (2.3)	24	24	0	Feeding study	DLW	Minnesota	MJ/day	C
Conway 2002 B						DLW	Tecumseh		
Conway 2002 AA	41.2 (2.0)	24	24	0	Feeding study	DLW	PAR	MJ/day	C
Conway 2002 BB						DLW	Stanford		
Davis 2004 A	19–69	62	24	38	Obese	Accel	YPAS	kcal/day	NS
Davis 2004 B						Accel		kcal/kg/day	
Davis 2004 C					Normal weight	Accel		kcal/day	
Davis 2004 D						Accel		kcal/kg/day	
Ekelund 2006 A	20–69	185	87	98	Swedish adults	Accel	IPAQ – MPA	min/day	A
Ekelund 2006 B						Accel	IPAQ – VPA		
Fogelholm 1998	29–46	20	0	20	Overwgt premenopause	HRM	PAD	min/day	A
Friedenreich 2006 A	35–65	154	75	79	Healthy Canadians	Accel	PAQ	MET-hr/week	B
Friedenreich 2006 B						Accel	7-day PAR	MET-hr/week	
Friedenreich 2006 C						Accel	PAQ	hr/week	
Friedenreich 2006 D						Accel	7-day PAR	hr/week	
Hagfors 2005	diet 58.8 (9.9), control 59.5 (8.1)	9	3	6	Rheumatoid Arthritis	DLW	3-day PAR	MJ/day	C
Hayden-Wade 2003 A	18–67	69	25	44	Active and sedentary	Accel	7-day PAR – MPA	min/week	A
Hayden-Wade 2003 B						Accel	7-day PAR – Hard		
Hayden-Wade 2003 C						Accel	7-day PAR – Vhard		
Iqbal 2006 A	26 (3.8)	50	0	50	Pakistani	Accel	MOSPA – LT	kcal/day	A
Iqbal 2006 B						Accel	MOSPA – ST		
Irwin 2001 A	27–65	24	24	0	Adult men	DLW	7-day PAR	kcal/day	C
Irwin 2001 B						DLW	PAL		
Jacobs Jr 1993 A	20–59	78	28	50		Accel	Minnesota	MET/min/day	NS
Jacobs Jr 1993 B						Accel	7-day PAR		
Jacobs Jr 1993 C						Accel	CAQ		
Jakicic 1998 A	25–50	50	0	50	Overwgt under reporters	Accel	PAR	min/week	A
Jakicic 1998 B					Overwgt over reporters	Accel	PAR		
Johnson Kozlow 2006 A	35–77	96	0	96	Breast cancer	Accel	IPAQ	min/week	A
Johnson Kozlow 2006 B		63	0	63			7-day PAR		
Johnson-Kozlow 2007	38–72	63	0	63	Breast cancer	Accel	WHIQ	min/week	A
Koulouri 2006 A	28.3 (6.0)	10			Healthy – baseline	Ped	PAD	KJ	A
Koulouri 2006 B					Healthy – intervention	Ped			
Lasuzzo 2004	71.7 (8.2)	39	19	20	Seniors	Accel	Fullerton	MET-min/day	A
Leenders 1998 A	18–40	10	0	10	Healthy females	Accel, HRM, DLW	7-day PAR	kcal/day	A
Leenders 1998 B						Accel, DLW	PAEE	kcal/day	
Leenders 2000	26 (6.6)	12	0	12	Healthy college	Accel, Ped	7-day PAR	kcal/kg/day	A
Leenders 2001	21–37	13	0	13	Healthy females	Accel, Ped, DLW	7-day PAR	kcal/day	A
Lemmer 2001 A	20–30	40	21	19	Younger males	Accel	PAQ	kJ/day	C
Lemmer 2001 B	65–75				Older males	Accel			
Levin 1999 A	21–59	77	28	49	Healthy volunteers	Accel	4-week PAR	MET-min/day	C
Levin 1999 B							SAFE		
Liu 2001	22–86	31			Elderly Chinese	Indirect cal.	PAQ	kcal	NS
Lof 2003 A	21–41	34	0	34	Healthy Swedish	Accel, HRM, DLW	PAQ – TEE	kJ/24-hour	A
Lof 2003 B						HRM	PAQ – PAEE		
Lovejoy 2001 A	47.4 (0.2)	149	0	149	Premenopausal African	Accel	PAQ	kJ/day	D
Lovejoy 2001 B					Premenopausal White	Accel		kJ/day	
Masse 1999	35–61	31	0	31	Volunteers	Accel	3-day PAD	kcal/day	A
Matthews 1995 A	26.7	25	14	11	University volunteers	Accel	3-day PAL	kcal/day	A
Matthews 1995 B							7-day PAR		
Matthews 2005 A	46	69			Volunteers	Accel	24-hr PAR	min/day	C
Matthews 2005 B						Accel		min/wk	
Matthews 2005 C						Accel	STAR Closed	min/day	
Matthews 2005 D						Accel		min/wk	
Matthews 2005 E						Accel	STAR Open	min/day	
Matthews 2005 F						Accel		min/wk	
McDermott 2000 A	67.2 (7.0)	41	19	22	Peripheral Arterial Disease	Accel	LTPAQ	kcal/week	A
McDermott 2000 B	66.1 (5.4)				Non-PAD	Accel			
Meriwether 2006 A	20–61	63	10	58	Volunteers	Accel	PAAT	min/week	A
Meriwether 2006 B						Accel	IPAQ		
Miller 2005 A	61.6 (7.2)	13			Diabetic/CABG intervention	Accel	7-day PAD	kcal/kg/day	D
Miller 2005 B					Diabetic/CABG control	Accel			
Paton 1996	26–45	10	10	0	HIV positive	DLW	PAD	PA Level	A
Paul 2005	39 (9)	12	12	0	Non-smoking	DLW	7-day PAQ	MJ/day	A
Pitta 2005 A	61 (8)	13	10	3	COPD – Walking	Accel	1-day PAD	mins.	A
Pitta 2005 B					COPD – Cycling	Accel			
Pitta 2005 C					COPD – Standing	Accel			
Pitta 2005 D					COPD – Sitting	Accel			
Pitta 2005 E					COPD – Lying	Accel			
Racette 1995 A	21–47	14	0	14	Obese	HRM, DLW	7-day PAR	MJ/day	C
Racette 1995 B						HRM		kcal/day	
Richardson 1994 A	20–59	78	28	50	Healthy	Accel	Minnesota	MET/min/day	C
Richardson 1994 B						Accel	4-week PAQ		
Richardson 1994 C						Accel	48-hr PAL		
Richardson 1995	20–59	78	28	50	Healthy	Accel	Baecke	MET/min/day	A
Richardson 2001	20–59	77	27	50	Healthy	Accel	Stanford 7-day PAR	MET/min/day	A
Rothenberg 1998	73	20	8	12	Healthy, free-living	HRM, DLW	PAD	MJ/day	C
Rutgers 1997 A	68–78	13	0	13	Elderly – individual calibration	HRM	PAQ	MJ/day	A
Rutgers 1997 B					Elderly – group calibration	HRM			
Schmidt 2003 A	39–65	58	0	58	College – Freedson	Accel	PAL	min/day	A
Schmidt 2003 B					College – Hendelman	Accel			
Schmidt 2003 C					College – Swartz	Accel			
Schmidt 2006 A	18–47	54	0	54	Pregnant – Freedson	Accel	PPAQ	MET-min/day	C
Schmidt 2006 B					Pregnant – Hendelman	Accel			
Schmidt 2006 C					Pregnant – Swartz	Accel			
Schulz 1989 A	20–30	6	4	2	Healthy university	HRM – Proc 1	PAD – Procedure 5	MJ/day	A
Schulz 1989 B						HRM – Proc 2	PAD – Procedure 5		
Schulz 1989 C						HRM – Proc 3	PAD – Procedure 5		
Schulz 1989 D						HRM – Proc 4	PAD – Procedure 5		
Schulz 1989 E						HRM – Proc 1	PAD – Procedure 6		
Schulz 1989 F						HRM – Proc 2	PAD – Procedure 6		
Schulz 1989 G						HRM – Proc 3	PAD – Procedure 6		
Schulz 1989 H						HRM – Proc 4	PAD – Procedure 6		
Schulz 1989 I						DLW	PAD – Procedure 5		
Schulz 1989 J						DLW	PAD – Procedure 6		
Seale 2002 A	67–82	27	14	13	Rural elderly	DLW	7-day PAR – PAWT	MJ/day	C
Seale 2002 B						DLW	7-day PAR – PABMR		
Seale 2002 C						DLW	7-day PAR – PAREE		
Sjostrom 2002 A	41 (10)	445	202	243	Swedish	Accel	IPAQ – MPA	min/day	A
Sjostrom 2002 B						Accel	IPAQ – VPA		
Sobngwi 2001	19–68	89	44	45	Cameroonian	Accel	SSAAQ	MET-hr/day	C
Soundy 2005 A	52.9 (9.0)	9			Severe mental illness	Accel	PAQ – week 2	kcal/day	C
Soundy 2005 B						Accel	PAQ – week 6		
Starling 1998	52–79	65	28	37	Older free-living	DLW	Minnesota	kcal/day	NS
Starling 1999 A	45–84	67	32	35	Older free-living	Accel, DLW	YPAS	kcal/day	NS
Starling 1999 B						Accel	Minnesota		
Staten 2001 A	31–60	35	0	35	Sedentary	DLW	AAFQ (28-day)	kJ/day	NS
Staten 2001 B						DLW	AAFQ (7-day)		
Stein 2003 A	30.6 (4.7)	56	0	56	Pregnant – active	Accel, HRM	PAL	kcal/day	A
Stein 2003 B	27.9 (5.4)				Pregnant – sedentary	Accel, HRM			
Strath 2003 A	20–56	25	12	13	General population	Accel	CAQ – MPA	min/day	A
Strath 2003 B						Accel	CAQ – VPA		
Strath 2003 C						Accel	CAQ – MPA+ VPA		
Strath 2004	20–65	15	12	14	General population	Accel	CAQ (with HR)	MET-min/wk	NS
Taylor 1984 A	34–69	12	12	0	White males	Accel	Stanford	kcal/day	NS
Taylor 1984 B						Accel		kcal/kg/day	
Taylor 1984 C						Accel	PAD	kcal/day	
Taylor 1984 D						Accel		kcal/kg/day	
Timperio 2003 A	M 37.8 (12.7),	122	55	63	Overweight	Accel	PAQ > 3 MET	min/day	A
Timperio 2003 B	F 39.6 (17.0)					Accel	PAQ 3–5.9 MET		
Timperio 2003 C						Accel	PAQ > 6 MET		
Timperio 2004 A	18–75	551	241	310	Free-living	Accel	AAS – Log	min/day	A
Timperio 2004 B						Accel	AAS – No log		
Timperio 2004 C						Accel	IPAQ-S – Log		
Timperio 2004 D						Accel	IPAQ-S – No log		
Timperio 2004 E						Accel	IPAQ-L – Log		
Timperio 2004 F						Accel	IPAQ-L – No log		
Timperio 2004 G						Accel	BRFSS – Log		
Timperio 2004 H						Accel	BRFSS – No log		
Tzetzis 2001	19–21	75	33	42	Novice skiers	HRM	PAQ	time/task	B
Wadsworth 2006 A	18–24	71	0	71	College – controls	Accel	IPAQ – MPA	min/day	A
Wadsworth 2006 B					College – controls	Accel	IPAQ – VPA		
Wadsworth 2006 C					College – intervention	Accel	IPAQ – MPA		
Wadsworth 2006 D					College – intervention	Accel	IPAQ – VPA		
Walsh 2004 A	20–46	75	0	75	Overweight White	DLW	Tecumseh	kcal/day	NS
Walsh 2004 B					Overweight Black	DLW			
Walsh 2004 C					Control White	DLW			
Walsh 2004 D					Control Black	DLW			
Washburn 2003	18–33	46	17	29	Sedentary mod obese	DLW	PAR	kJ/day	C
Wendel-Vos 2003 A	44 (6)	50	36	14	Healthy bank workers	Accel	PAQ 2–4 MET	MET	D
Wendel-Vos 2003 B						Accel	PAQ 4–6.5 MET		
Wendel-Vos 2003 C						Accel	PAQ 6.5+ MET		
Wickel 2006	18–23	70	13	57	University	Accel	PAD	kcal/day	A
Wilbur 2001 A	45–65	156	0	156	Healthy employees	HRM	PAQ	min/walk	A
Wilbur 2001 B						HRM	PAL		

* A – measuring same length of time (e.g. 7-days, 1 week) and no time lag, B – same length with time lag, C – different length with no time lag, D – different length with a time lag, NS – not stated

Abbreviations: AAFQ – Arizona Activity Frequency Questionnaire, AAS – Active Australia Survey, Accel. – accelerometer, BRFSS – Behavioral Risk Factor Surveillance System, Cal. – calorimetry, CABG – Coronary Artery Bypass Graft, CAQ – College Alumnus Questionnaire, COPD – chronic obstructive pulmonary disease, DLW – doubly labeled water, F – female, Fullerton – Fullerton Physical Activity Questionnaire, HRM – heart rate monitor, IPAQ – International Physical Activity Questionnaire, kcal – kilocalorie, kJ – kilojoules, KPAS – Kaiser Physical Activity Survey, LTPAQ – Leisure Time Physical Activity Questionnaire, M – male, MAQ – Modifiable Activity Questionnaire, MET – metabolic equivalent, MJ – mega joules, min – minute, Minnesota – Minnesota Leisure Time Physical Activity Questionnaire, MOSPA – Monica Optional Study of Physical Activity questionnaire, MPS – moderate physical activity, PAAT – Physical Activity Assessment Tool, PAD – physical activity diary, PAEE – physical activity energy expenditure, PAFQ – Physical Activity Frequency Questionnaire, PAL – physical activity log, PAQ – physical activity questionnaire, PAR – physical activity recall, Ped. – pedometer, SAFE – Survey of Activity, Fitness, and Exercise, SSAAQ – Sub-Saharan Africa Activity Questionnaire, Stanford – Stanford Physical Activity Questionnaire, STAR – Short Telephone-administered Activity Recall, Tecumseh – Tecumseh Community Health Survey, TEE – total energy expenditure, Vhard – very hard, VPA – vigorous physical activity, WHIQ – Women's Health Initiative Questionnaire, wk – week, YPAS – Yale Physical Activity Survey

Data abstraction identified three articles and two dissertations that analyzed and reported duplicate data in multiple papers [184-188]. Authors of suspected duplications were contacted and in cases where several publications reported the same analyses from the same data source, only one study per data source/analysis was retained in order to avoid double counting. Studies were retained based on the most pertinent and most recent data, as well as the largest sample size. Studies included were published over a 24-year period from 1983 to 2007. All studies were written in English. Nineteen of the studies used randomized controlled trial designs [22,24,26,28,30,50,53,61,84,91,124,148,149,163,165,171,181] and all others used observational designs (e.g. case control, cross-sectional, longitudinal). All included studies were published as journal articles except for 19 dissertations [16,24,30,34,38,45,49,61,64,69,71,73,74,78,99,107,117,163,171].

Participants in the studies ranged from 10 to 101 years of age. Although the focus of the review was on those aged 18 and over, studies that had a range of ages less than 18 years were not excluded as long as the mean age of the sample was over 18 years. Sample sizes ranged from a low of six [21] to a high of 2,721 in Craig *et al.*'s work that assessed the validity of the International Physical Activity Questionnaire (IPAQ) [35]. There were a greater number of studies reporting on female-only data than studies reporting on male-only data.

A total of five direct measures were used in the assessment of physical activity and included: accelerometers, DLW, indirect calorimetry, HRM, and pedometers. Of the studies included in the synthesis of directly comparable data (Table 2), accelerometers were the most frequently used direct measure and indirect calorimetry was the least used. A variety of self-report measures were employed, but the seven-day physical activity recall (7-day PAR) [189] was the most cited. Over half of the studies reported that the self-report and directly assessed physical activity levels were measured over the same length of time (e.g. seven days) and over the same period of time (i.e. no time lag between measurements). There were also a considerable number who reported measurements over the same period of time, but that did not measure the same length of time (e.g. self-report over seven days, directly measured over three days). Eleven of the studies in Table 2 lacked any mention of time [59,131,135,138,143,159,160,164,177,178,183].

### Risk of bias assessment

Risk of bias was assessed for all included studies (n = 173) including those reporting only correlation data. The range of items met on the modified Downs and Black tool was 8 to 15 (maximum possible count was 15) with a mean of 11.7 ± 1.2. Results of the risk of bias assessment indicated that 38% (65/173) of the studies had lower quality (based on a median split count of < 12/15). All studies were given maximum points for describing study objectives. All but one study scored maximum points for describing the main outcomes to be measured and the interventions used (including comparison methods between measures). Although most studies carried out some sort of significance testing on results, most did not report the actual probability values associated with the estimates or their associated measures of random variability (e.g. standard error or confidence intervals). Most studies obtained a high number of items on the reporting section (maximum count of 8) with a mean of 6.9 ± 0.9.

The external validity section of the risk of bias assessment had a maximum count of three and consisted of reporting on the representativenessof the subjects and the testing conditions. Almost all of the studies (166/173) reported that the staff, places and facilities where the participants were tested were representative of the testing conditions that would be expected by most individuals (e.g. real-life and free-living situations). However, 87% (151/173) of the studies did not report on the representativeness of the subjects asked to participate in the study and 95% (165/173) of the studies failed to report on the representativeness of those who were prepared to participate (enrolled) compared to the entire population from which they were recruited (received a score of 0). As a result, the external validity ratings of most studies were poor with a mean of 1.1 ± 0.5.

In order to obtain the maximum number of items (four) in the internal validity section, studies must have reported whether any of the results of the study were based on "data dredging", whether the analyses adjusted for any time lag between the two measurements or different lengths of follow-up, whether the statistical tests used to assess the main outcomes were appropriate, and whether the main outcome measures were accurate (valid and reliable). Internal validity item counts were generally high with the majority of studies having obtained a four.

A qualitative analysis was conducted on the top seven (scores of 14 and 15 out of 15) and lowest seven studies (8 and 9 out of 15) based on scores from the risk of bias assessment. No conclusive patterns were identified from this analysis. The results from the accelerometer studies were further examined, as this was the only group of studies with a good distribution of low and high quality studies based on the accelerometer median split of bias scores. Findings from this analysis did not identify any clear patterns in the differences in agreement between physical activity measured by self-report compared to accelerometer when grouped by low and high quality.

### Data synthesis

**Figure 2:**
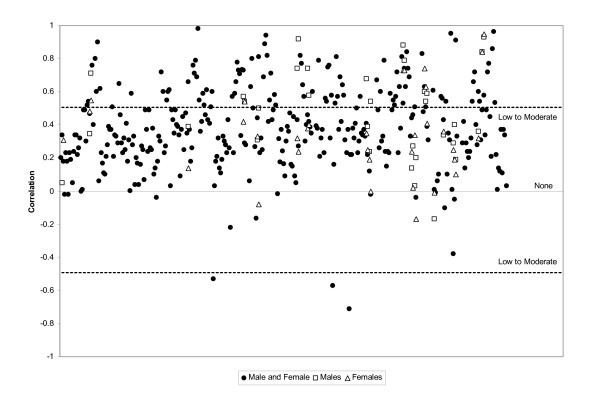
Scatter plot of all correlation coefficients between direct measures and self-report measures.

One hundred and forty-eight studies [10,11,13-157,190] reported correlation statistics between self-report and direct measurements of physical activity. Figure 2 is a plot of all extracted correlations and shows that overall, there is no clear trend in the degree of correlation between self-reported and directly measured physical activity, regardless of the direct method employed. Overall, correlations were low-to-moderate with a mean of 0.37 (SD = 0.25) and a range of -0.71 to 0.98. Mean correlations were higher in studies reporting results for males-only (r = 0.47) versus studies reporting results for females-only (r = 0.36), but with very similar ranges (males: -0.17 to 0.93 vs. females: -0.17 to 0.95).

**Figure 3:**
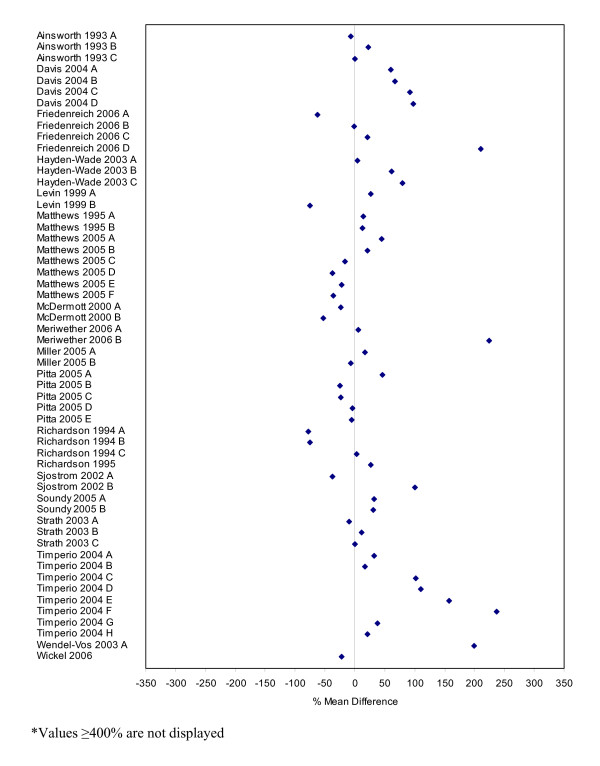
Forest plot of percent mean differences between accelerometers and self-report measures from studies reporting combined results for males and females (excluding outliers ≥ 400%).

**Figure 4:**
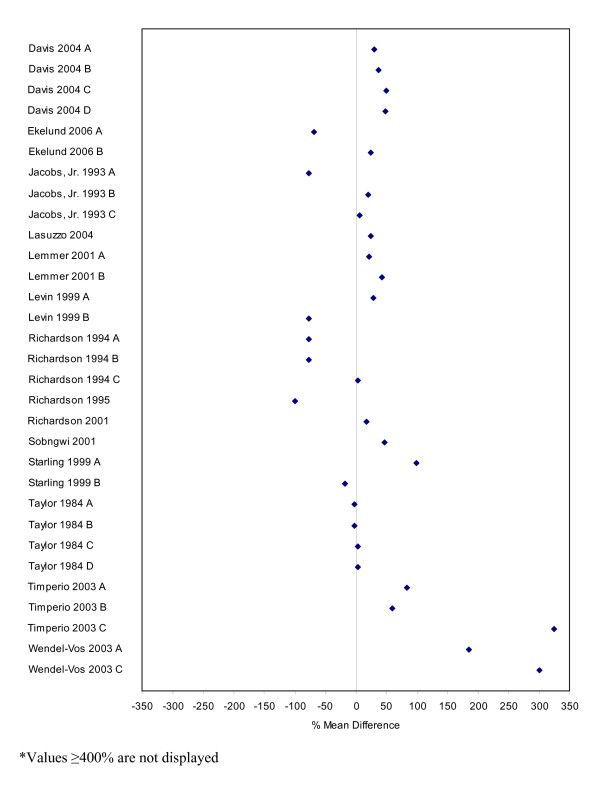
Forest plot of percent mean differences between accelerometers and self-report measures from studies reporting results for males only (excluding outliers ≥ 400%).

**Figure 5:**
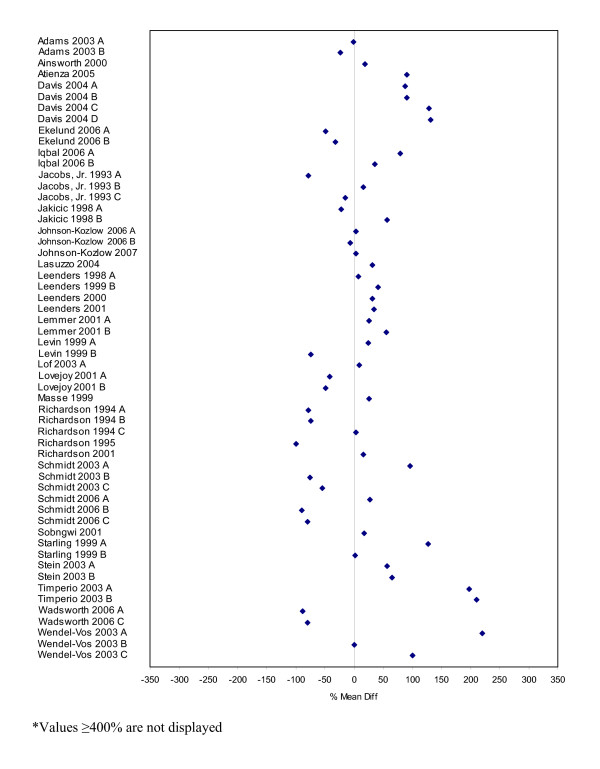
Forest plot of percent mean differences between accelerometers and self-report measures from studies reporting results for females only (excluding outliers ≥ 400%).

**Figure 6:**
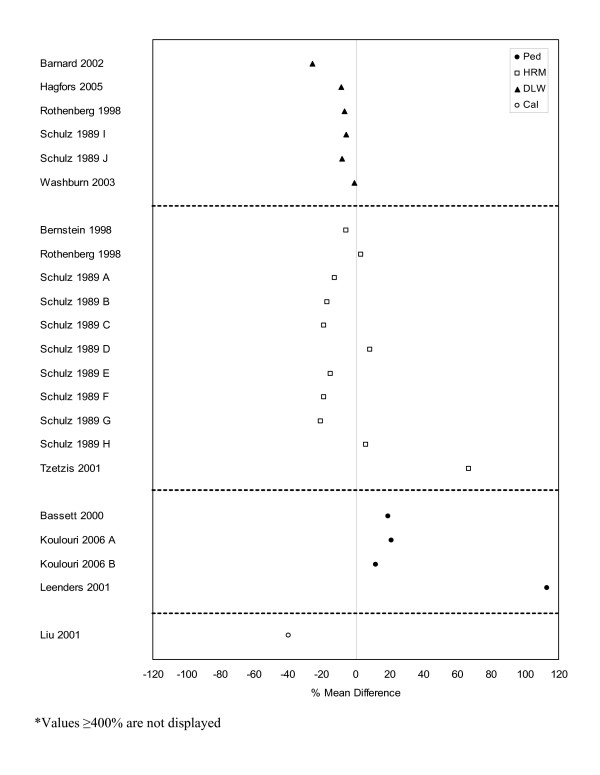
**Forest plot of percent mean differences between doubly-labeled water, heart rate monitoring, pedometers, and indirect calorimetry and self-report measures from studies reporting combined results for males and females (excluding outliers ≥ 400%). **Cal – calorimetry, DLW – doubly labeled water, HRM – heart rate monitor, Ped – pedometer.

**Figure 7:**
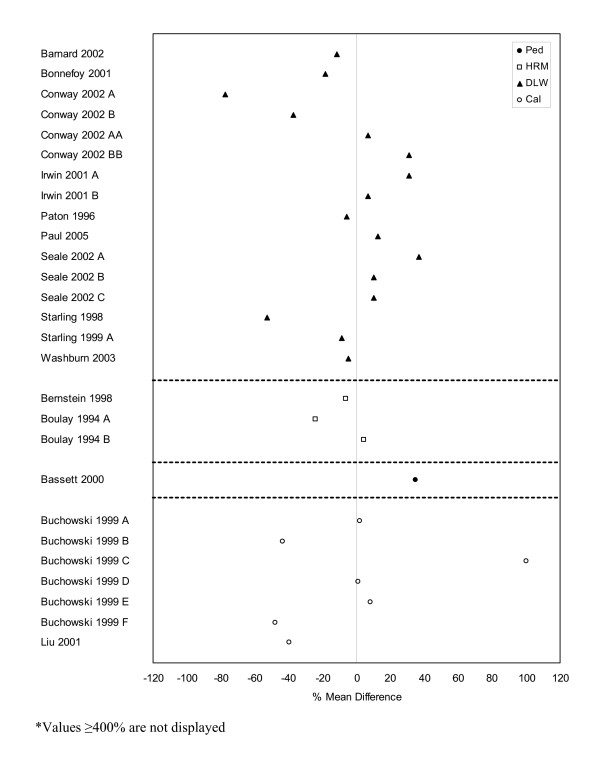
**Forest plot of percent mean differences between doubly-labeled water, heart rate monitoring, pedometers, and indirect calorimetry and self-report measures from studies reporting results for males only (excluding outliers ≥ 400%).** Cal – calorimetry, DLW – doubly labeled water, HRM – heart rate monitor, Ped – pedometer.

**Figure 8:**
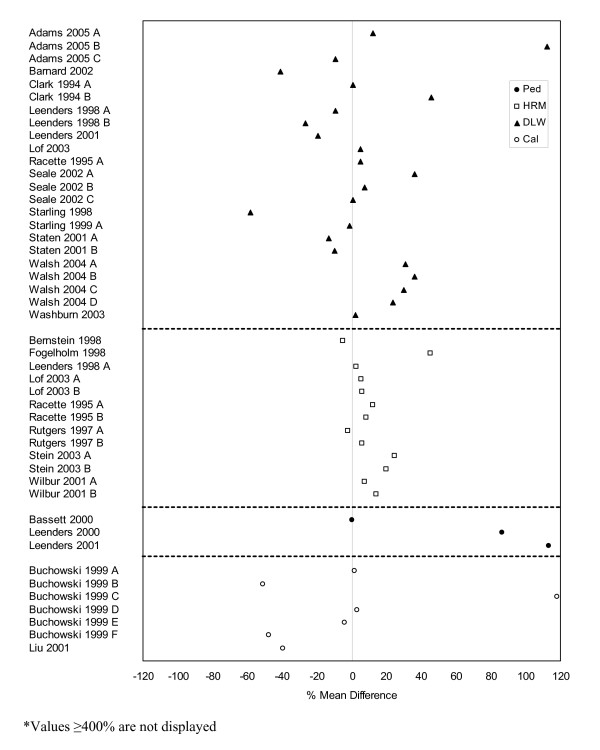
**Forest plot of percent mean differences between doubly-labeled water, heart rate monitoring, pedometers, and indirect calorimetry and self-report measures from studies reporting results for females only (excluding outliers ≥ 400%). **Cal – calorimetry, DLW – doubly labeled water, HRM – heart rate monitor, Ped – pedometer.

Seventy-four studies contained comparable data on the measurement of physical activity based on self-report and directly measured values. Table 2 describes these studies and their subcomponents. Percent mean differences were calculated for all of these studies and are presented as forest plots in Figures 3 to 8. Negative values indicate that self-report estimates were lower than the amount of physical activity assessed by direct methods while positive values indicate values that are higher. Sixty percent of the percent mean differences indicated that self-reported physical activity estimates were higher than those measured by direct methods.

Studies with extreme percent mean differences (≥ 400%) were removed from the forest plots for clarity purposes [11,139,151,181]. All outlying data were from studies where physical activity was categorized by level of exertion (e.g. easy, moderate, vigorous) and outliers represent physical activity data categorized as vigorous or of high energy expenditure. While not all data categorized as vigorous had percent mean differences ≥ 400%, a pattern emerged whereby greater percent mean differences between the self-report and direct measures was larger for vigorous levels of physical activity than for light or moderate activities [11,44,56,134,139,151,175,181,182].

Percent mean differences were examined separately for the five different direct measures. Accelerometers were the most used direct measure. Self-report measures of physical activity were generally higher than those directly measured by accelerometers (Figures 3 to 5). Studies reporting data for males and females combined (n = 58) had a mean percent difference of 44% (range: -78% to 500%), with similar findings for the male-only data (n = 32) (mean: 44%, range: -100% to 425%). However, female-only data (n = 60) identified that, on average, females self-reported higher levels of physical activity compared to accelerometers with a mean percent difference of 138% (range: -100% to 4024%).

The second-most common direct measure employed was DLW and comparable data with self-report measures are presented in Figures 6 to 8. Studies reporting on combined male and female data (n = 6) indicated that self-report measures of physical activity were lower when compared to DLW measures with a mean percent difference of -9% and a range of -1% to -26%. Results for male-only (n = 16) and female-only (n = 23) data were less distinct with percent mean differences and ranges of -4.5% (-78% to 37%) and 7% (-58% to 113%), respectively.

A greater number of HRM and self-report comparisons were observed for studies with both male and female participants (n = 11) or female-only populations (n = 13) versus male-only populations (n = 3). Female-only results showed a general trend toward higher levels of self-reported physical activity (mean 11%, range: -5% to 45%), while the male-only (mean -9%, range: -24% to 5%) and combined (mean -2%, range: -21% to 67%) data had a greater number of studies with lower self-reported physical activity levels when compared to results of HRM.

Pedometers and indirect calorimetry were the least commonly used direct measures for studies with comparable data. There were a total of eight comparisons from four studies for pedometers and 15 from two studies for indirect calorimetry (Figures 6 to 8) making it difficult to draw conclusions with regard to patterns of agreement between the self-report and direct measures. However, seven [19,75,76,167] of the eight pedometer comparisons reported higher levels of physical activity by self-report when compared to the pedometer results. The eighth comparison [19] which involved female-only data saw no difference between the two measures. The indirect calorimetry results were less straightforward and presented no obvious patterns in agreement.

Subgroups were qualitatively examined to assess whether any differences existed in the degree of agreement between self-reported and directly measured physical activity. No clear patterns emerged within studies reporting on elderly (range or mean ≥ 65 years) populations [23,73,77,92,105,116,174] or within studies reporting on different time lags and periods of measurement. Few studies with comparable data reported exclusively on overweight/obese populations, but amongst those captured, the majority of studies reported higher levels of physical activity by self-report compared to the direct measures [139,143,148,163-165,172]. However, it was not possible to compare the overweight/obese percent mean differences to those reported in general populations.

Meta-analyses were not possible due to the substantial heterogeneity in units of reporting for physical activity measured by the various self-report and direct methods across the studies, and the significant lack of data with comparable units across measures. As a result, we were unable to determine the sensitivity of the values and the associated measures of error for the studies. Overall effect sizes to summarize the magnitude of discrepancy across the various measures of physical activity could therefore not be calculated.

## Discussion

To the authors' knowledge this review represents the most comprehensive attempt to examine the relationship between self-report and directly measured estimates of adult physical activity in the international literature. Risk of bias was assessed and identified that just over one third of the studies had lower quality based on their description of the methods and external and internal validity. Overall, no clear trends emerged in the over- or underreporting of physical activity by self-report compared to direct methods. However, some results suggest that patterns in the agreement between self-report and direct measures of physical activity may exist, but they are likely to differ depending on the direct methods used for comparison and the sex of the population sampled. Interestingly, findings also identified that studies which categorized physical activity by level of exertion (e.g. light, moderate, vigorous) exhibited a trend wherein these categorized studies saw the mean percent differences between the self-report and direct measures increasing with the higher category levels of intensity (i.e. vigorous physical activity). These larger differences may reflect a problem with self-report measures attempting to capture higher levels of physical activity, or problems with participant interpretation and recall.

Many of the studies tested the relationship between self-report and direct measures by using a correlation coefficient, but this is limited as correlation is only able to measure the strength of the relationship between two variables and cannot assess the level of agreement between them, as well as ignoring any bias in the data [191]. A more useful approach, the Bland-Altman method, provides a means for assessing the level of agreement between self-report and direct measures by deriving the mean difference between the two measures and the limits of agreement. If the two measures possess good agreement and measure the same parameter of physical activity, then the cheaper and less invasive self-report methods may be valid substitutes for direct methods.

A meta-analysis would have allowed us to estimate the overall effect sizes for each of the direct measures and undertake a sensitivity analysis to further understand the degree of bias in the studies. Unfortunately, inconsistent methods and reporting among the studies included made such an analysis methodologically inappropriate. Further research in this area would benefit from greater consistency in the units of reporting and the methods used to facilitate comparisons. For instance, many studies did not report results using the same units, so estimates of agreement between the self-report and direct measures could not be computed. There was also an inconsistency in the number of days measured and the time lag between the self-report and direct measures. It is recommended that authors present their results using the same units for both measures (e.g. minutes/day, kcal/day), that the two measurements assess physical activity for and over the same time period, and that all relevant data including a mean and measurement of variance (i.e. standard deviation, standard error) be included in all reports.

Adhering to consistent reporting criteria would increase the comparability of results across studies and enable the calculation of overall effect sizes. At the population level, over- or underestimation of physical activity prevalence has important implications as these data are used to monitor physical activity trends, determine spending for research and physical activity interventions and programming, and to estimate physical inactivity-related risks of disease. Future studies may wish to refer to the updated Compendium of Physical Activities [192] which provides a coding scheme to classify physical activity by rate of energy expenditure. The Compendium offers a means to increase the comparability of results between self-report and direct measures, as well as across studies.

A lack of a clear trend amongst the differences between the self-report methods for assessing physical activity and the more robust direct methods is of concern, especially when trying to establish whether the measures could be used interchangeably. There are several possible explanations for the lack of a clear trend in the data. Many self-report instruments (such as the 7-day PAR) may not have the ability to account for activities of less than 10 minutes in duration or those with a level of exertion lower than brisk walking [193], whereas some of the direct methods (such as DLW) may capture all forms of physical movement. However, it is important to recognize that other direct measures such as accelerometers are unable to capture certain types of activities such as swimming and activities involving the use of upper extremities. Our findings demonstrate the inherent difficulty self-report measures possess when trying to accurately capture data at various levels of exertion. Compared to direct measures, self-report methods appear to estimate greater amounts of higher intensity (i.e. vigorous) physical activities than in the low-to-moderate levels.

Just as with some self-report measures not being able to capture all forms of activity, some direct measures may capture non-physical activity. For instance, the DLW technique is an accurate assessment of total energy expenditure, but it does not only capture physical activity, but rather all forms of energy expenditure including resting energy expenditure and the thermogenic effect of food. DLW is therefore expected to overestimate physical activity unless corrections are made. These and other measurement errors may inflate the between-individual variability in the energy expended in physical activity [194]. Finally, direct methods may be too sensitive to small errors derived from the various calibration methods employed and the equations used to define and categorize physical activity.

It is important to take into account all of these factors when comparing self-report and direct measures of physical activity. In specific circumstances (e.g. at different levels of activity) these two methods may not be comparable as they are not able to capture the same parameters of physical activity. Self-report measures may not able to accurately capture all levels of activity, but they may be able to capture how difficult an individual perceives an activity to be and the type of activity that is undertaken (e.g. leisure, work, transportation). Direct measures, on the other hand, may be more able to capture some of the information not captured in self-report methods (e.g. incidental daily movement and lower intensity activities), but also possess their own limitations such as the inability to capture arm movements and various types of physical activity (e.g. swimming).

Concern regarding the discrepancy between self-reported and directly measured physical activity were recently reported by Troiano and colleagues who examined data from the 2003–2004 National Health and Nutrition Examination Survey (NHANES) which contained the first direct measurements of physical activity in a nationally representative U.S. sample [195]. They compared self-reported adherence estimates of physical activity recommendations with those directly measured by accelerometer. Their findings identified that self-reported adherence estimates were much higher than those measured by accelerometer. The authors hypothesize that the overestimation may be a result of respondents misclassifying sedentary or light activity as moderate or from underestimations of activity duration by the accelerometers.

Other factors, such as those related to the population under study, may influence the ability of self-report and direct methods to capture the same measurement. For example, our findings show that in studies with a focus on overweight/obese individuals, self-reported physical activity was overestimated in all cases except for DLW studies involving combined male/female and male-only data. Our results differed from those reported by Irwin, Ainsworth and Conway (2001) [58]. Their study consisted of 24 males and used DLW to compare energy expenditure estimates with those obtained by physical activity record and the 7-day PAR. The investigators observed an overestimation of energy expenditure in participants with higher body fat using the physical activity record, but not the 7-day PAR. A comparison of the same sample by body mass index (BMI) identified that those with a BMI ≥ 25 kg/m^2 ^overestimated energy expenditure from physical activity records and the 7-day PAR. In confirmation of the trends within our accelerometer data, a recent study (published after our search) of 154 subjects compared a physical activity questionnaire to accelerometry data and identified that the accuracy of the physical activity questionnaire was higher for males than females and for those with a lower BMI [196]. It is likely that a response bias exists due to social desirability, and influences the degree of over-reporting of physical activity by overweight/obese individuals. Future research and synthesis is needed to identify whether a bias does in fact exist and if so, whether it differs by gender, and to what extent.

This review had limitations that should be considered when examining the results. First, the sample was limited to studies that included directly comparable data between self-report and direct measures (same units for both measures) or a comparison by way of correlation. Access to primary data from each study was not feasible; therefore, we relied upon reported comparisons and the means of measured physical activity. This reduced the number of studies with reported measures of physical activity by self-report and direct methods and limited our ability to accurately assess the degree of agreement between the two measures. However, when possible we converted non-comparable units to increase the number of studies used. The review did not assess the agreement between proxy-reported physical activity and direct measures. Proxy-report data are less prevalent but is an important means for assessing physical activity in sub-populations such as those who are chronically ill, disabled, or elderly, and who are unable to self-report on their own physical activity levels. Further research is required to assess the validity of proxy-report measures of physical activity when compared to direct methods. Finally, this review did not discern between differences in study protocols related to calibration, cut-points, or collection of the measurements and other population specific characteristics.

## Conclusion

In conclusion, this review provides an objective summary of the difference in physical activity levels assessed via self-report methods compared to directly measured physical activity. The results may assist researchers considering the use of self-report or direct measurement methods and serves as a note of caution that self-report and directly measured physical activity can differ greatly. Overall there were no clear trends in the degree to which physical activity measured by self-report and direct measures differ. The strength of trends differed by the direct method employed and by the gender of the population sampled. One-third of the studies were of poor quality with most studies having failed to report actual probabilities or measures of variability for estimates and the representativeness of their samples. The costs and benefits of direct measurement need to be considered in any study in order to determine if the added resources required for personnel training and laboratory analyses justify the possible increase in the precision of results. At this time, it is not possible to draw any definitive conclusions concerning the validity of self-report measurements compared to various direct methods, but caution should be exerted when comparing studies across methods.

## Competing interests

The authors declare that they have no competing interests.

## Authors' contributions

SAP carried out the design, bibliographic search, article screening, data abstraction and synthesis and drafted and edited the manuscript. KBA participated in its design and coordination and helped edit the manuscript. MH participated in article screening, data abstraction and editing of the manuscript. JH participated in data abstraction, data synthesis and helped edit the manuscript. SCG participated in the design of the study, provided methodological input, and assisted in the editing of the manuscript. MT conceived the study, and participated in its design and coordination and helped to draft the manuscript. All authors read and approved the final manuscript.
